# Organic Disordered Semiconductors as Networks Embedded in Space and Energy

**DOI:** 10.3390/nano12234279

**Published:** 2022-12-01

**Authors:** Lucas Cuadra, Sancho Salcedo-Sanz, José Carlos Nieto-Borge

**Affiliations:** 1 Department of Signal Processing and Communications, University of Alcalá, 28801 Alcalá de Henares, Spain; 2 Department of Physics and Mathematics, University of Alcalá, 28801 Alcalá de Henares, Spain

**Keywords:** disordered organic semiconductors, hopping transport, variable-range hopping, space–energy embedded networks

## Abstract

Organic disordered semiconductors have a growing importance because of their low cost, mechanical flexibility, and multiple applications in thermoelectric devices, biosensors, and optoelectronic devices. Carrier transport consists of variable-range hopping between localized quantum states, which are disordered in both space and energy within the Gaussian disorder model. In this paper, we model an organic disordered semiconductor system as a network embedded in both space and energy so that a node represents a localized state while a link encodes the probability (or, equivalently, the Miller–Abrahams hopping rate) for carriers to hop between nodes. The associated network Laplacian matrix allows for the study of carrier dynamics using edge-centric random walks, in which links are activated by the corresponding carrier hopping rates. Our simulation work suggests that at room temperature the network exhibits a strong propensity for small-network nature, a beneficial property that in network science is related to the ease of exchanging information, particles, or energy in many different systems. However, this is not the case at low temperature. Our analysis suggests that there could be a parallelism between the well-known dependence of carrier mobility on temperature and the potential emergence of the small-world property with increasing temperature.

## 1. Introduction

Organic semiconductors (OSCs) are currently attracting a huge amount of attention worldwide because of their intrinsic beneficial properties such as flexibility (crucial in wearable electronics and flexible organic solar cells [1]), their feasibility for controlling their molecular design [2], and, what is of essential importance, their low cost. In-depth knowledge and the ability to perform accurate simulations [3] of carrier transport in OSCs are key for increasing the performance of organic thermoelectric devices [4,5], organic photovoltaic cells [6,7], organic light-emitting diodes (OLEDs) [8,9], organic thin-film transistors (OTFTs) [10,11], and organic field-effect transistors (OFETs) [12]. Specifically, as the OSC channel is very sensitive to exogenous stimulus, OFETs can be used as sensors for a wide variety of physical variables [13,14]. Additionally, two-dimensional (2D) OSC nanostructures are highly sensitive to bio-analytes and have a bio-functionality which assists in designing more efficient bio-sensors [15] and other nanodevices [16].

In particular, an important subset of OSCs is the one made up of organic disordered semiconductors (ODSs), including conjugated polymers, low-molecular-weight materials, and doped polymers [17]. Regarding this, most scientists and engineers agree that the transport of electric charge is mainly based on carrier hopping between localized quantum states, which are disordered in both space and energy [17,18,19,20,21,22,23,24]. This has been experimentally tested in [18], and proven by both analytical models [17,20,22,23,24,25,26,27] and numerical simulations [3,19,21,28,29,30].

**Figure 1:**
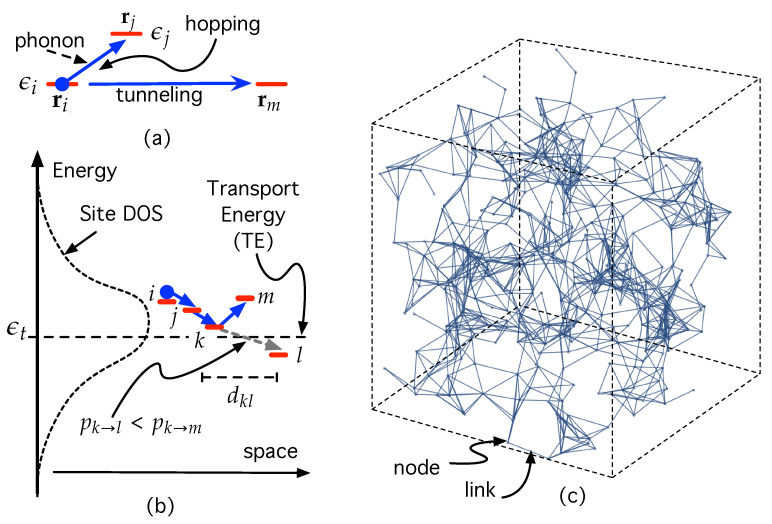
(**a**) Illustration of VRH concept. (**b**) Qualitative representation of how carriers hop between states whose DOS is Gaussian. $\varepsilon_{t}$ stands for the transport energy. See the main text for details. (**c**) Network representing a three-dimensional ODS sample. Each node encodes a localized state (site), while each link represents a possible carrier hopping between sites.

In turn, hopping transport in ODSs belongs to a complex class of hopping called variable-range hopping (VRH) [31,32,33]. This means that, as shown in Figure 1a, a carrier in an initial state *i* (with energy $\varepsilon_{i}$ and located at site $r_{i}$$\in R^{3}$) may either jump to a near state *j* at $r_{j}$ with different energy, $\varepsilon_{j}$ (thanks to phonon absorption), or may tunnel to a further state *m* with the same energy $\varepsilon_{i}$ [16]. The Miller–Abrahams (MA) [34] and the Marcus [35] models are used profusely to compute carrier hopping rates between full and empty states. The latter is the most widely used in electron transfer involving electrochemical processes in molecular chemistry and biology [36,37,38,39,40]. The MA model is the most used in the field of ODSs because it is easier to apply and leads to very accurate results in most cases [17,20,22,23,24,25,26,27,31]. In the present work, we use hopping rates inspired by the MA model. As will be shown later on, the carrier hopping probability involving states *i* and *j* is proportional to (∝), a negative exponential that depends on both the spatial Euclidean distance $d_{E,ij}$ in $R^{3}$ and an energy distance function $\varepsilon_{ij}$

(1) $$p_{ij}\propto \exp\left(-\frac{2d_{E,ij}}{\xi_{\mathrm{loc}}}\right)\cdot \exp\left(-\frac{\varepsilon_{ij}}{k_{B}T}\right),$$

where $\xi_{\mathrm{loc}}$ is the carrier localization length and $k_{B}T$ is the thermal energy

Thus, not only does the site spatial distribution in $R^{3}$ play a key role but so does the energy spectrum in the density of states (DOS). Although there has been some controversy about the DOS in ODSs (either exponential or Gaussian), it has been recently shown that the DOS in ODSs is Gaussian [17]. The reason why an exponential DOS is sometimes used is because it helps simplify the study of hopping transport, leading to an exact solution [17,41]. In the present work, we also use a Gaussian DOS within the framework stated by the Gaussian disorder model (GDM) [17,21] to achieve a sufficiently realistic modeling.

As shown in Figure 1b, we have also represented a threshold energy level, $\varepsilon_{t}$, called transport energy (TE) [17,26,42], which separates two different types of hops between states with different energy. On the one hand, if the carrier occupies a state with energy $\varepsilon >\varepsilon_{t}$, then the transport consists of hops downwards in energy. On the other hand, if the carrier occupies a state with energy $\varepsilon <\varepsilon_{t}$, then transport occurs by an activated hop (phonon absorption) upwards in energy. Note that the energy contribution in Equation (1) vanishes when $\varepsilon_{i}>\varepsilon_{j}$. These is because in cases in which an empty state *j* is near *i* energy, downward transitions are much more likely than the corresponding upwards transitions because the latter require the concurrence of phonons. Nevertheless, at some point *k* during the carrier path shown in Figure 1b, the distance between state *k* and another *l* with $\varepsilon_{l}<\varepsilon_{k}$, $d_{kl}$ may be so large that it is more likely for the carrier to be pumped by a phonon up to a more energetic state *m* with $\varepsilon_{m}>\varepsilon_{t}$. That is, $p_{k\to l}<p_{k\to m}$. This is only the physical meaning of the TE; it has a characteristic energy level $\varepsilon_{t}$ that separates low energy states $\varepsilon_{l}<\varepsilon_{t}$ that are so far apart in space that it is more likely to be a carrier–phonon interaction that promotes the carrier up to a state with $\varepsilon_{m}>\varepsilon_{t}$.

Complementary to the concept of TE, hopping is also studied by combining VRH with the percolation concept (see [41] and the references therein for details). The basic idea of the percolation approach, which is applicable in many fields of science [43], consists of searching for the critical value of a magnitude that allows the emergence of connectivity between the constituents of a network [44,45,46], or undergo a phase transition [43]. Indeed, the percolation approach for modeling VRH has been profusely exploited [47,48,49,50,51]. It was used for studying hopping transport between states with exponential DOS in [49] and [50], although with different percolation criteria [41]. More recently, the percolation theory has been explored combining VRH and the GDM in [42].

The complex dependency of VRH on both space and energy within the framework of GDM is precisely what compels us to model ODSs as a space–energy embedded network.

Network science (NS) [52] has become a successful, multidisciplinary approach that allows the study of many different systems, both natural and artificial. All of them have in common the fact that they consist of a large number of interacting elements that can be represented using a network (or, mathematically, a graph) [53], that is, a collection of “nodes” (or “vertices”) attached by “links” (or “edges”). Simply put, a node represents an interacting element of a system that is connected to others by means of a relationship (human networks) or by the exchange of particles ([54,55] in nanostructures), energy (in electric grids [56]) or information (communication networks [57]). Thanks to this versatility, NS allows for understanding the structure and behavior of systems showing very different natures [44,52,58,59], involving both artificial systems (blockchain [60], electric grids [56,61,62], the Internet [63], transport networks [64]), natural systems (the emergence of interstellar molecular complexity [65], complex Earth systems [66], the human brain [67], ecosystems [68], vascular networks [69], and metabolic networks [70]. More examples can be found in [52,58,71] and the references therein. Furthermore, NS math tools [72,73] also assist in understanding epidemic processes [74] such as the proliferation of COVID-19 [75], the chain of successive collapses in artificial networks [76,77], or the dissemination [78,79] and persistence of information, memes, or ideas [80].

The purpose of this paper consists of modeling an ODS system as a network embedded in both space and energy so that a node represents a confined state (see Figure 1c) while a link encodes the probability (or, equivalently, the rate) for a carrier to hop from one state (=node) to another, taking into account the VHR within the GDM.

We have organized the paper as follows. Section 2 shows the ODS system to be studied and the method by which we generate the corresponding network embedded in both space and energy. Section 3 contains our simulation work. This suggests that, at room temperature, the network exhibits a strong propensity for a small-network nature, a beneficial property which, as will be demonstrated later on, has been found to enhance the exchange of information [81] (social networks [82], human brain [83,84]); matter (electrons in quantum dot systems [54,55] sap in vascular networks in plants [85]); and energy (in power grids [61,62]) in the field of network science. The results suggest that there could be a parallelism between the well-known dependence of carrier mobility on temperature and the potential emergence of the small-world property with increasing temperature. Finally, Section 4 summarizes the model and the most important concussions.

## 2. Proposed Model: The ODS System and its Associated Network

### 2.1. The ODS System

Consider a three-dimensional sample of ODS that has a density of localized states $N_{S}$, which are disordered in both space and energy. In space, each localized state is centered on a site characterized by a position vector $r_{i}$$\in R^{3}$. Each site is randomly distributed in three-dimensional space. Furthermore, each of these localized states is characterized by a random energy according to an energy spectrum given by a Gaussian DOS [17,26],

(2) $$g\left(\varepsilon \right)=\frac{N_{S}}{\sigma \sqrt{2\pi }}\exp\left(-\frac{\varepsilon^{2}}{2\sigma^{2}}\right),$$

where σ is the *energy scale* of the DOS and $N_{S}$ is the concentration of confined states. Typical values for these parameters are σ≈0,1 eV, while $N_{S}$ may range from $N_{S}\approx 10^{20}$ cm${}^{-3}$ to $N_{S}\approx 10^{21}$ cm${}^{-3}$ [17]. The carrier hopping model through confined states that are placed at random in space and have the Gaussian energy spectrum stated by Equation (2) is the GDM [17,26] we mentioned in Section 1.

In thermal equilibriums, the average rate transition for carriers between a localized state *i* (located at $r_{i}$ and characterized by an energy $\varepsilon_{i}$) and another state $j(r_{j},\varepsilon_{j})$ is, according to the MA hopping model [17,26,34,47]

(3) $$\Gamma_{ij}=\gamma_{0}\exp\left(-\frac{d_{E,ij}}{\xi_{\mathrm{loc}}/2}-\frac{|\varepsilon_{i}-\varepsilon_{j}\left|+\right|\varepsilon_{i}-E_{F}\left|+\right|\varepsilon_{j}-E_{F}|}{2k_{B}T}\right),$$

where $E_{F}$ is the Fermi level of the carrier concentration *n* and $\gamma_{0}$ is the attempt-to-escape frequency. It depends on the interaction with phonons, and its value is usually assumed to be $\gamma_{0}\approx 10^{12}$ s${}^{-1}$ [20,86]. In Equation (3), $\xi_{\mathrm{loc}}$ is in the order of magnitude $\xi_{\mathrm{loc}}\geq 10^{-8}$ cm [87]. According to the detailed balance principle, $\Gamma_{ij}=\Gamma_{ji}$, as clearly explained in [47]. Note that the energy-dependent function $\varepsilon_{ij}$ in Equation (1) is

(4) $$\varepsilon_{ij}=\frac{|\varepsilon_{i}-\varepsilon_{j}\left|+\right|\varepsilon_{i}-E_{F}\left|+\right|\varepsilon_{j}-E_{F}|}{2},$$

the energy-dependent part of the MA-based rate Equation (3).

The relative concentration (carriers/sites) in the experiments that follow is $n/N_{S}=10^{-4}$. At this low carrier concentration *n*, carriers behave independently from each other [17].

### 2.2. Defining the Network Associated with the ODS System

When aiming to map the ODS system to a network embedded in space–energy, it is necessary to properly identify nodes and links.

Each quantum-localized state |i〉 is represented as a node *i* in the network. Any node *i* is characterized by two parameters: its position vector $r_{i}$$\in R^{3}$ and its energy $\varepsilon_{i}$. As site concentration in the ODS system is $N_{s}$, then the number of nodes is $N=N_{s}V$, where *V* is the volume of the ODS sample at hand.

While identifying nodes has seemed pretty intuitive (|i〉↔ site $i(r_{i},\varepsilon_{i})$↔ node *i*), the method by which links are defined requires specific knowledge of ODSs within the GDM. We consider that a link between two nodes *i* and *j* is formed with probability

(5) $$p_{ij}=\exp\left(-\frac{2d_{E,ij}}{\xi_{\mathrm{loc}}}-\frac{|\varepsilon_{i}-\varepsilon_{j}\left|+\right|\varepsilon_{i}-E_{F}\left|+\right|\varepsilon_{j}-E_{F}|}{2k_{B}T}\right)\equiv \exp\left(-{\tilde{d}}_{S-E}\right),$$

which satisfies $p_{ij}=\Gamma_{ij}/\gamma_{0}$, as stated by Equation (3). ${\tilde{d}}_{S-E}$ represents a normalized space–energy distance in the networks at hand.

At this point, we need to use some network science concepts. The first of these is the concept of the adjacency matrix, A, which represents the direct connection (by exchanging a charge carrier = link) between any two pairs of nodes *i* and *j*: $a_{ij}=1$ or $a_{ij}=0$. A gives an idea of the structural connectivity of a network. Sometimes, this binary information is not enough if, for example, we want to study the carrier dynamics. In this effort, we associate a weight to each link, obtaining a weighted adjacency matrix W [71] Our adjacency matrix is an N×N matrix with elements

(6) $${\left(W\right)}_{ij}=\left\{\begin{array}{cc} 0 & ,\mathrm{if}\;i=i\\ \Gamma_{ij} & ,\mathrm{if}\;i\neq j, \end{array}\right.$$

where $\Gamma_{ij}$ is given by Equation (3).

Thanks to these matrices, the system is represented by a network whose graph is G≡G(N,L,W), where N is the set of nodes (card(N)=N) and L is the set of links. Note that as a consequence of the method by which this network has been generated, it contains information on not only both the location and energy level of the sites but also on the corresponding carrier hopping rates.

## 3. Simulations: Experimental Work

### 3.1. Methodology

Let us consider a three-dimensional sample of ODS that has a density of localized states $N_{S}$, which are disordered in both space and energy as described in Section 2.1. The sites are randomly distributed in the three-dimensional space, according to a uniform distribution U(a,b), with $a=2\xi_{loc}$ and $b=L_{\mathrm{sample}}=100\times \xi_{loc}$, being $L_{\mathrm{sample}}$ the size of the ODS sample. Each of these localized states *i* has an energy level $\varepsilon_{i}$ belonging to the Gaussian DOS stated by Equation (2) within the GDM framework [17,26]. As the site concentration in the ODS system is $N_{S}$, then the number of nodes is $N=N_{S}V$, where $V={\left(L_{\mathrm{sample}}\right)}^{3}$, the volume of the ODS sample at hand. As we proposed in Section 2.2, we represent any localized quantum state |i〉 (at $r_{i}$ and with energy $\varepsilon_{i}$) as a node labeled *i*↔ site $i(r_{i},\varepsilon_{i})$↔|i〉 in the network. Each link between nodes *i* and *j* represents a carrier hopping between them with probability $p_{ij}$ given by Equation (5).

Aiming to obtain statistical values, we generated 50 different realizations for any network with a number of nodes *N*.

### 3.2. Exploring Carrier Dynamics

The weighted adjacency matrix W stated by Equation (6) can be used to help us compute the so-called Laplacian matrix L [73,88], which in turn allows for studying carrier dynamics using stochastic random walks (RWs). The reason for using RWs is because the transport is *incoherent* as a consequence of carrier–phonon interactions (emission or absorption of phonons in each hop), causing the charge carrier to lose its phase information [32]. The Laplacian matrix is defined as L=D−W [89], D being the diagonal matrix whose elements $D_{i}=\sum_{i\neq j}{\left(W\right)}_{ij}$ are the hopping rate strength of node *i*.

The questions arising here are: How does L help compute the carrier dynamics? What is the probability for a carrier initially localized at node *j* (state or |j〉) to hop to another node *k* (=|k〉) after a time *t*: $p_{kj}\left(t\right)$? The answer is well known in the field of networks. As shown in [89,90,91], a “walker” (the charge carrier in our system) performs a RW according to the equation

(7) $$\frac{d}{dt}p_{kj}\left(t\right)=-\sum_{m}{\left(L\right)}_{km}p_{mj}\left(t\right).$$

whose formal solution is [90]

(8) $$p_{kj}\left(t\right)=\langle k|e^{-Lt}|j\rangle =\sum_{n}e^{-\lambda_{n}t}\langle k|q_{n}\rangle \langle q_{n}|j\rangle ,$$

where $\lambda_{n}$ are the eigenvalues of L, which are real numbers and fulfill $\lambda_{n}\geq 0$. In the class of RWs associated with the Laplacian L called “edge-centric RW” [89], any link in a node *i* is generating ruled by the carrier hopping rate $\Gamma_{ij}$. Once a link i↭j is activated, the carrier behaves as a random walker. Such a random walk model is also called the “fluid model” [89] and means in NS that a carrier (walker) goes out of a node *i* characterized with a high strength $D_{i}=\sum_{i\neq j}{\left(W\right)}_{ij}$ (that is, with higher rates) faster than from other node with lower rate sum.

A useful related parameter that can give us an idea of the global carrier hopping efficiency is the so-called average return probability (ARP) [90]

(9) $$\bar{p}\left(t\right)=\frac{1}{N}\sum_{j}p_{jj}\left(t\right).$$

Its physical meaning is as follows. A high value of $\bar{p}\left(t\right)$ means that the hopping is not efficient because the particle has a high probability of staying at the starting node [92]. However, a low value of $\bar{p}\left(t\right)$, approaching unity from below, suggests that the carrier can quickly hop from node to node during the time interval *t*. We thus define the hopping transport efficiency (HTE) as [54]

(10) $$\eta_{HT}\left(t\right)=1-\bar{p}\left(t\right).$$

**Figure 2:**
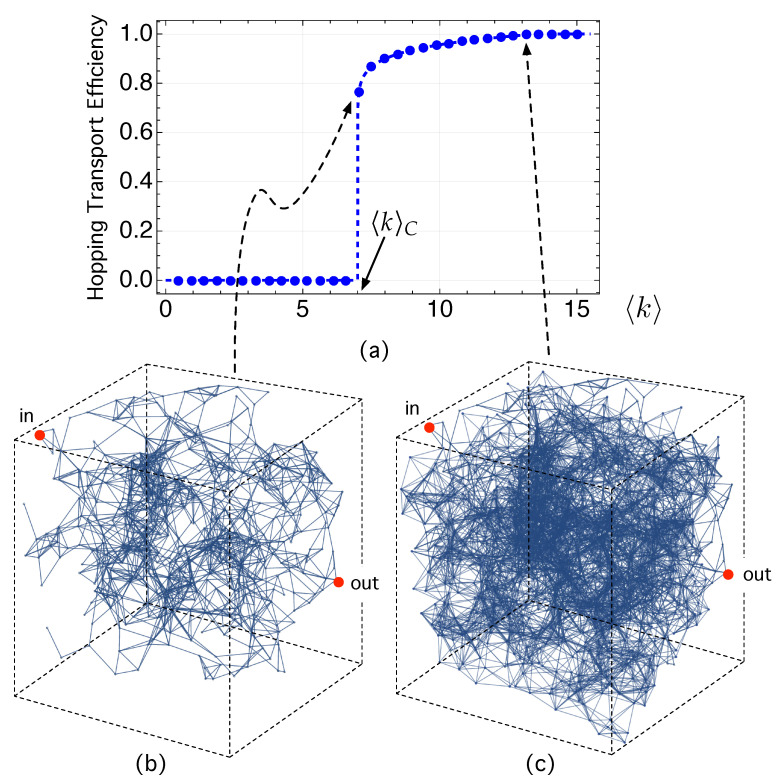
(**a**) Hopping transport efficiency, averaged over time, as a function of the average node degree 〈k〉. (**b**) Minimal sub-network (or infinite clustering) for which a carrier in node “in” on the left side of the sample is able to reach the opposite side in node “out”. The rest of the smaller sub-networks disconnected from each other have not been drawn for the sake of clarity. (**c**) Complete network with N=1000 nodes that contains the critical sub-network illustrated in (**b**). See the main text for the corresponding discussion.

In precisely this respect, Figure 2 shows the hopping transport efficiency as a function of the mean degree 〈k〉, *k* being the degree or number of links of a given node.

The hopping transport efficiency, that is, the extent that a carrier can hop through the network, depends on the distance in space and energy among the different nodes. When the site density is too small, the nodes (= sites) are so far apart that $\exp(-2d_{E,ij}/\xi_{\mathrm{loc}})\to 0)$ in Equation (5). As a consequence, $p_{ij}\to 0$ and no link can be formed: 〈k〉=0. As all nodes are completely isolated, then a carrier remains localized, is not allowed to hop, and thus HTE =0.

As the node *N* increases, the localized states become closer and closer, leading to the formation of links among groups of nodes called clusters. Thus, the mean degree becomes (〈k〉>0). The clusters that are initially formed are small and disconnected to each other. These disconnected sub-networks, known as components, initially show a similar size.

As 〈k〉 increases, one of the clusters begins to connect with others, becoming larger and larger. This largest sub-network is known as a giant component in NS. Note that, at ${\langle k\rangle }_{C}\approx 7$, the emergence of this largest sub-network (when compared to the others) also makes the HTE have an abrupt change: while HTE(〈k〉)=0 for 〈k〉<${\langle k\rangle }_{C}\approx 7$, however, HTE changes abruptly and reaches the value HTE$({\langle k\rangle }_{C})\approx 0.78$. This is because this dominant component is also the minimum sub-network (“infinite cluster” in material science) or critical sub-network (see Figure 2b) for which a carrier in the red node labeled “in” on the left side of the sample is able to reach the opposite side at node “out”. This is a percolation transition. The HTE parameter becomes its order parameter. Following [45] we have denoted it as m(〈k〉). According to [45], the explored network shows a hybrid percolation transition because it combines, at the same point ${\langle k\rangle }_{C}$, features of both first-order phase transition (a very fast change of the order parameter) and second-order phase transition (critical phenomena). The order parameter, m(〈k〉)≡ HTE (〈k〉) fulfills

(11) $$\mathrm{HTE}\left(\langle k\rangle \right)=\left\{\begin{array}{cc} 0 & ,\mathrm{if}\langle k\rangle <{\langle k\rangle }_{C}\\ {\mathrm{HTE}}_{0}+z\cdot {\left(\langle k\rangle -{\langle k\rangle }_{C}\right)}^{\beta_{HTE}} & ,\mathrm{if}\langle k\rangle \geq {\langle k\rangle }_{C}, \end{array}\right.$$

where ${\mathrm{HTE}}_{0}$ and *z* are constants and $\beta_{HTE}$ is the critical exponent of the order parameter. For 〈k〉>${\langle k\rangle }_{C}=7$, HTE$\left(\langle k\rangle \right)\approx 0.4+0.9{\left(\langle k\rangle -7\right)}^{0.08}$.

Finally, note in Figure 2c that the complete network with 〈k〉≈13 shows HTE =1 because the carrier can potentially hop across all nodes of the network. The HTE corresponding to the critical sub-network is less than unity because a carrier may become trapped in some of the remaining smaller isolated sub-networks that, for illustrative purposes, we have not drawn in Figure 2b.

### 3.3. Studying the Network Structure: Navigating the Network

A concept that emerged from NS which has long fascinated the general public is the “six degrees of separation” theory [93,94], which provides an idea of how surprisingly easy communication can be on a network. The concept grew out of some social experiments whose objective was to determine the average number of times a letter had to be sent in order for it to reach a person in another city. Such a number was found to be six, that is, an average distance of six. Today’s huge social networks, with billions of connections, can have average distance between users as small as 4.74 [82]. The shortest path between two nodes *i* and *k* is the minimum number of hops for walking from node *i* to *k*. Its mean value over the whole network, the “average shortest path length” *ℓ* [52], suggests the ease of navigating the network, hopping from node to node as if the network were “small”. The fact that *ℓ* scales logarithmically with the network size (number of nodes, *N*) [52], ℓ∼lnN, is one of the features of “small-world” networks. Another feature is a high “mean clustering coefficient”. A high clustering means a high density of triangles in the sense that when two nodes are linked to a third one, they usually tend to have a high probability of being linked to each other. The clustering coefficient of node *i* with $k_{i}$ links is the ratio between the number of links that exist between the $k_{i}$ involved, $M_{i}$, and the maximum number of links: $C_{i}=2M_{i}/\left(k_{i}\left(k_{i}-1\right)\right)$ [52]. The mean clustering coefficient over the complete network is thus $\langle C\rangle =\frac{1}{N}\sum_{i}C_{i}$.

As suggested, the most striking idea of small-world networks characterized by high local clustering and short average shortest path between any two nodes is that even though they can be made up of a huge number of interacting nodes, they nonetheless greatly enhance the exchange of information [81] (social networks [82], human brain [83,84]); matter (electrons in quantum dot systems [54,55], sap in vascular networks in plants [85]); or energy (in power grids [61,62]) between the involved nodes. However, as discussed in [95], it is necessary to quantify the shortness of *ℓ* is and the height of 〈C〉. This could be the case for the proposed network under certain circumstances.

To quantify whether or not our network is a small-world network, we have made use of a new metric, the small-world propensity (SWP), recently proposed in [95]. It aims to measure the extent that the mean clustering coefficient and the short average shortest path ( 〈C〉, *ℓ* ) deviate from those of equivalent random networks ($C_{R}$, $L_{R}$) and lattice networks ($C_{L}$, $L_{L}$) [95]. The SWP is defined as [95]

(12) $$\mathrm{SWP}≐1-\left(\frac{\Delta_{C}^{2}+\Delta_{L}^{2}}{2}\right),$$

where $\Delta_{C}=\frac{C_{L}-\langle C\rangle }{C_{L}-C_{R}}$ and $\Delta_{L}=\frac{\ell -L_{R}}{L_{L}-L_{R}}$. As discussed in [95], networks with high small-world features (low $\Delta_{C}$ and $\Delta_{L}$) will have a value of the SWP close to 1. The authors have chosen a threshold value of 0.6 to differentiate a network with strong small-world propensity from a network with weak small-world propensity.

**Figure 3:**
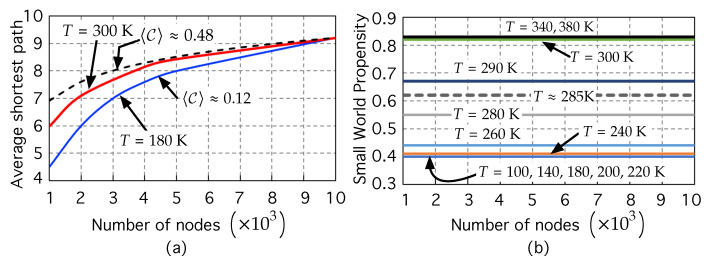
(**a**) Average shortest path as a function of the number of nodes of the network, for two different limiting temperatures, 180 K and 300 K. Any point on the lines is the mean value computed over 50 realizations of each network to obtain accurate statistical values. The dotted black line represents lnN for comparative purposes. (**b**) small-world propensity, stated by Equation (12) as a function the number of nodes. It has been computed for different values of increasing temperature *T*.

Figure 3a shows the average shortest path lengths *ℓ* (over 50 realizations) of different space–energy embedded networks (generated by the method proposed in Section 2.2) as a function of the network size, which range from $N=10^{3}$ to $N=10^{4}$ nodes.

For illustrative purposes, we have represented the average shortest path length *ℓ* for two different temperatures that, as will be explained later on, are representative of two classes of networks (small-world networks and non-small networks).

The red line corresponds to the results computed at room temperature T=300 K, while the blue line corresponds to those computed at a lower, representative temperature, T=180 K. Additionally, we have also plotted the dotted black line lnN for comparative purposes. Figure 3a shows that both curves scale with *N* with a tendency slightly lower than lnN, which gets closer and closer to lnN as *N* approaches $10^{4}$ nodes. One might think that this is indicative of a small-world network. However, this is not enough, and even could be misleading. Note that the clustering coefficients are very different in both cases. At low temperature, the clustering coefficient is small 〈C〉≈0.12. However, at room temperature, the clustering coefficient is high 〈C〉≈0.48. The network at room temperature appears to include the two typical ingredients of the small world: short *ℓ* (scaling ℓ∼lnN) and high 〈C〉. However, what do “short” and “high” mean here?

To discern whether or not the small-world feature exists we have used the SWP metric [95], represented in Figure 3b, as a function of the number of nodes.

For low temperatures (T= 100, 140, 180, 200, and 220 K, in our simulations, to save computational time), the networks have an SWP that remains constant: SWP ≈0.4. This means that in this low temperature range there is a clear “*non-*small-world regime”. At the other extreme of temperatures explored, T= 285, 290, 300, 340, 380 K, the networks have SWP > SWP${}_{Th}=0.6$, and thus are in the “small-world regime”. At the “representative” room temperature (T= 300 K), the network exhibits a high SWP value of ≈0.82, much higher than the threshold SWP${}_{Th}=0.6$ established in [95]. That is, at room temperature, the space–energy embedded network associated with the ODS system shows a strong small-world propensity: low $\Delta_{C}$ and $\Delta_{L}$.

In between these two regimes, Figure 3b shows that there is an “intermediate regime” in which, as temperature rises (T= 240, 260, 280, 285 K), the SWP starts to grow first slowly (T= 240, 260 K) and then faster (T= 280, 285 K), approaching the threshold value SWP${}_{Th}=0.6$ from below. This seems to suggest the emergence of the small-network nature.

Regarding this, the question that now arises is whether or not this has any influence on the charge carrier hopping transport in ODSs.

### 3.4. Carrier Mobility and Network Structure

Assisted by Figure 1b, we have mentioned that hopping transport in the GDM for ODSs consists of downward transitions in energy for carriers with $\varepsilon >\varepsilon_{t}$, and phonon-assisted upward transitions, for carriers with $\varepsilon <\varepsilon_{t}$ [17,26,42]. $\varepsilon_{t}$ is the energy value that optimizes the hopping rates in Equation (3) with respect to energy, including its percolation origin and the dependence of carrier concentration on the Fermi level $E_{F}$ [42,96]. Once $\varepsilon_{t}$ is obtained, the carrier mobility, for low carrier concentration (relative concentration $n/N_{S}\leq 10^{-4}$), can be approximated by [42,97,98]

(13) $$\mu \approx \mu_{0,LCC}\cdot \exp\left(-\frac{2r(\varepsilon_{t})}{\xi_{\mathrm{loc}}}-\frac{\varepsilon_{t}}{k_{B}T}-\frac{1}{2}{\left(\frac{\sigma }{k_{B}T}\right)}^{2}\right),$$

where $r(\varepsilon_{t})$ is the typical distance between localized states with energies $\varepsilon <\varepsilon_{t}$.

The network model we suggest in the present work allows for obtaining an estimation of the value of $r(\varepsilon_{t})$ based on the average space–energy distance between the network nodes having an energy distance function $\varepsilon_{ij}$ (Equation (4)) approaching $\varepsilon_{t}$. In the set of simulations described in Section 3.3 we have found that the estimated value $\tilde{r}\left(\varepsilon_{t}\right)$ seems to have the trend

(14) $$\tilde{r}\left(\varepsilon_{t}\right)\propto \xi_{\mathrm{loc}}\frac{\varepsilon_{t}}{k_{B}T}.$$

We interpret this dependency as follows: as *T* rises, the increasing number of available phonons allows for hops that are further away in energy but closer in space, making $\tilde{r}\left(\varepsilon_{t}\right)$ decrease inversely proportional to *T*. Including this in Equation (15), we obtain the approximated equation for the hopping mobility in the proposed network framework as

(15) $$\mu \approx \mu_{0,LCC}^{*}\cdot \exp\left(-A^{*}{\left(\frac{\sigma }{k_{B}T}\right)}^{2}\right).$$

The coefficient $A^{*}$ that appears in Equation (15) has a slight dependence on the site concentration $N_{S}$ and the localization length $\xi_{\mathrm{loc}}$, as shown in [97]. To take this into account, we have considered two sets of simulations that differ in the value of the $N_{S}\xi_{\mathrm{loc}}$ parameter. The first set corresponds to $N_{S}\xi_{\mathrm{loc}}=0.001$. When analyzing the results we have obtained that is $A^{*}\approx 0.47$. The second set of simulations corresponds to $N_{S}\xi_{\mathrm{loc}}=0.025$. In this case, the value of $A^{*}$ coefficient reduces to $A^{*}\approx 0.40$. This decreasing trend agrees with the results obtained in [87,97,99] using analytical methods. In these works, the coefficient $A^{*}$ is called *C* and its analytical estimates have been found to be C≈0.46 (at $N_{S}\xi_{\mathrm{loc}}=0.001$) and C≈0.38 (at $N_{S}\xi_{\mathrm{loc}}=0.02$). We are aware that this difference is probably caused by numerical effects associated with the finite size of the explored network. Our result has been obtained with a network made up of $N=10^{4}$ nodes, which is the largest size that we have been able to simulate because of our computational limitations. However, it has been proven [100] that this order of magnitude of *N* is sufficient in the energy scale of 0.1 eV, the one considered in our simulations. In any case, Equation (15) exhibits the usual behavior of ODSs with Gaussian DOS [17] $\ln\left(\mu /\mu_{0,LCC}^{*}\right)\propto T^{-2}$.

**Figure 4:**
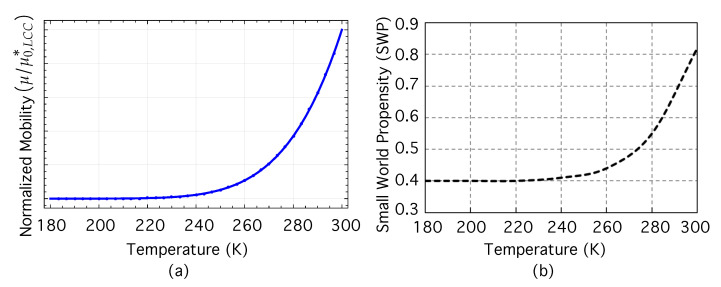
(**a**) Normalized mobility $\mu /\mu_{0,LCC}^{*}$ as a function of temperature *T* (K) for low carrier concentration (relative concentration $n/N_{S}\leq 10^{-4}$). (**b**) small-world propensity as a function of *T*(K). The number of network nodes is $N=10^{4}$. See the main text for further details.

Using Equation (15), Figure 4a shows the normalized mobility $\mu /\mu_{0,LCC}^{*}$ as a function of the increasing temperature *T*(K). Figure 4b, in turn, represents the dependence of the SWP on *T*. In these figures, we have selected the temperature interval 180 K ≤T≤ 300 K to show better the “intermediate regimen” (230 K ≤T≤ 285 K) in which the small-world nature begins to emerge. From the viewpoint of NS, the small-world property is related to the ease with which information, matter, or energy flows between nodes. In our problem, there seems to be a concordance between the increase in hopping carrier mobility with temperature (Figure 4a) and the growth of SWP with temperature (Figure 4b).

To discuss this further, let us return to Figure 3b, showing the SWP at different temperatures. For low temperatures (T<220), the networks have SWP ≈0.4, and are in the no small-world regime. For high temperatures (T>285), the networks have SWP > SWP${}_{Th}=0.6$, and are in the small-world regime. In between these two intervals, there is an intermediate regime in which, as temperature rises from 230 to 285 K, the SWP increases, approaching the threshold value SWP${}_{Th}=0.6$ from below. A transition to the small world seems to emerge in 230 K <T< 285 K. If we now turn our attention back to Figure 4a, it can be seen that normalized mobility has already begun to rise appreciably as temperature rises from ≈230 up to ≈280–290 K. In parallel we can observe in Figure 4b that as the temperature approaches ≈285 K from below, the network exceeds the threshold and begins to exhibit a strong small-world propensity. It seems to be a parallelism between the well-known dependence of carrier mobility on temperature $\left(\ln(\mu /\mu_{0,LCC}^{*}\propto T^{-2}\right)$ and the potential emergence of the small-world property with increasing temperature.

We realize that studying a system as extremely complex as an ODS through a network—an alternative mathematical representation that we build by selecting some of its properties (the random distributions of sites in space and energy together with the carrier hopping rates in the GDM model) to the detriment of others—could be argued to be a reductionist approach [56,101]. We have shown in Section 1 that there are many works that show how NS predicts collective emerging phenomena that are difficult or even impossible to explain based only on the properties of the elements that make them up. NS is simply a different, complementary approach, which can be used in parallel with other well-established methods, and does not intend to and cannot replace the other successful methods that are applied in materials science and nanotechnology.

**Figure 5:**
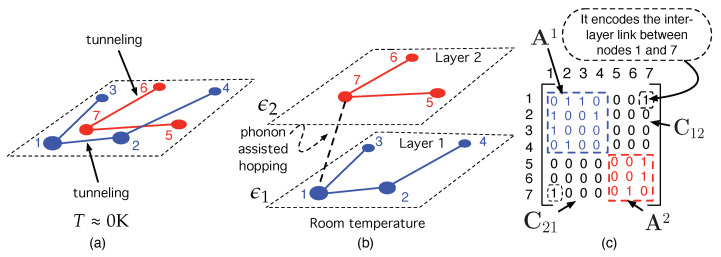
(**a**) A network with sites randomly distributed in space at very low temperature. Blue nodes have energy $\varepsilon_{1}$ while the red ones, energy $\varepsilon_{2}$. (**b**) Example of the emerging multilayer network as *T* increases. Each layer corresponds to a spatial network whose nodes have the same energy. (**c**) Corresponding adjacency matrix. See the main text for further details.

Related to this complementary character of the NS, we can consider a final example to illustrate the versatility of NS. Imagine a simple system that is made up of seven sites (=nodes) that are placed at random in space, as shown in Figure 5a. The blue nodes have an energy $\varepsilon_{1}$ while the red nodes have a different energy $\varepsilon_{2}$, slightly higher than $\varepsilon_{1}$. Because T≈0 K, only tunneling between sites with the same energy is allowed. Blue nodes may have some links depending on their distance (Equation (5)). Nearby red nodes may also have some links. However, there are no links between node 1 (blue, energy $\varepsilon_{1}$) and 7 (red, energy $\varepsilon_{2}$) because, despite being very close in space, they have different energy and, as there is no phonon available, then $p_{1,7}\ll p_{1,2}$.

However, as *T* increases, phonon-assisted transition is allowed, and the network in Figure 5a can now be unfolded into two layers, each corresponding to an energy level, as shown in Figure 5b. At room temperature, node 1, which is spatially very close to node 7, is now linked to node 7 (dotted line) because now there is phonon absorption and $p_{1,7}>p_{1,2}$. This is a toy example of a “multilayer network” [102]. Each layer is a network with some properties. In our example, a layer is a network formed by all nodes with the same energy. Figure 5b represents this concept. The first layer represents a network with $N_{1}=4$ blue nodes with energy $\varepsilon_{1}$ while the second one contains a network with $N_{2}=3$ red nodes with energy $\varepsilon_{2}$. The complete two-layer network has $N_{1}+N_{2}=7$ nodes.

The corresponding binary adjacency matrix of the two-layer network, represented in Figure 5c is made up of the sub-matrices $A^{1}$ (the one corresponding to the network in layer 1), $A^{2}$ (the adjacency matrix for the network in layer 2), and $C_{12}$ and $C_{21}$. These are matrices that encode the inter-layer connections between the networks elements in layers 1 and 2. In our example, there is only a link between node 1 in layer 1 and node 7 in layer 2 (Figure 5b). Its corresponding matrix element in $C_{12}$ has been marked in Figure 5c.

## 4. Summary and Conclusions

This paper proposes to model organic disordered semiconductors (ODSs) as a network embedded in both space and energy because ODSs are disordered not only in space but also in energy, which makes carrier transport more complex than in ordered semiconductors. Despite not being crystalline materials, ODSs have more and more practical importance because they have mechanical flexibility and a low cost, which is crucial for manufacturing wearable electronics and flexible solar cells. Additionally, because it is feasible to control the design of their constituent molecules, ODSs can be used to manufacture not only high-quality bio-sensors but also organic thermoelectric devices, organic thin-film transistors, and organic light-emitting diodes. The not-so-positive characteristic of ODSs is that charge carrier transport is difficult to model because it consists of carrier hops between localized quantum states, which are disordered in both space and energy. Furthermore, modeling is even more complex because hopping transport in ODSs is a variable-range hopping (VRH) in which a carrier in an initial state *i* (with energy $\varepsilon_{i}$ and located at site $r_{i}$$\in R^{3}$) may either hop to a near state *j* at $r_{j}$ and with different energy $\varepsilon_{j}$ (via phonon interaction) or may tunnel to a farther state *m* with the same energy $\varepsilon_{i}$. This hopping, involving confined states having a Gaussian density of states, is called Gaussian disorder model (GDM). In thermal equilibriums, the average carrier rate transition between two states *i* and *j* is modeled using Miller–Abrahams (MA) hopping rates $\Gamma_{ij}$, which are proportional to a negative exponential that includes both the spatial distance and an energy-difference function (temperature dependent) between the states involved.

An ODS can be thus seen as a complex system made up of a huge number of molecules, states, or sites ($N_{S}\approx 10^{20}$ cm${}^{-3}$) that interact with each other by exchanging charge carriers according to MA hopping rates. It is precisely this abstraction that compels us to use network concepts. Network science can be applied to any system made up of many elements that interact with each other exchanging information, energy, or particles. The essential principle is to map the system into a network (graph) in which any interacting element is represented by a node (vertex) and the interaction between them by a link (edge).

Specifically, in the proposed network, each quantum-localized state |i〉 in the ODS system is represented as a node *i* in the network. Note that each has two attributes: location $r_{i}$$\in R^{3}$ (space embedding) and energy $\varepsilon_{i}$ taken from a Gaussian distribution (energy embedding). In turn, a link between two nodes *i* and *j* is activated according to the probability $p_{ij}$ for a carrier to hop between them (or, equivalently, by the MA hopping rate $\Gamma_{ij}$). The matrix containing these rates is the weighted adjacency matrix W that, in turn, helps obtain the Laplacian matrix L. In NS, L is especially useful because it allows, among other features, to study the random walk (RW) of a carrier hopping through network nodes. The reason why we use RWs is because the transport is incoherent due to the carrier–phonon interaction, which causes the carrier to lose its phase information. In particular, the proposed network Laplacian matrix allow for the studying of carrier dynamics using edge-centric random walks, in which links are activated by the corresponding carrier hopping rates.

As a methodological approach aiming to obtain statistical values, we have generated 50 different realizations for any network with a number of nodes *N*.

We have built sets of spatial energy embedded networks with a number of nodes that ranges from $N=10^{3}$ to $N=10^{4}$ nodes. In turn, each of these networks has been generated at different temperatures (T= 100, 140, 180, 200, 220, 240, 260, 280, 285, 290, 300, 340, 380 K). As the link generation is ruled by carrier rates (which in turn depend on *T*), the networks obtained at each temperature, even having the same number of nodes, can be very different.

In fact, they are so different that the results seem to suggest that networks at room temperature exhibit a small-world nature, while at low temperatures, this does not occur. From the NS perspective, the small-world property is related to the ease with which information, matter, or energy flows between nodes. What should be checked in our problem is if this supposed structure enhances carrier transport. To quantify whether or not our explored networks have the small-world property, we have made use of a recently developed metric, the small-world propensity (SWP), which ranges between 0 and 1. The larger the SWP metric is, the more likely it is that the network is a small-world network. There is a threshold value, SWP${}_{Th}=0.6$, to differentiate a network with strong small-world propensity from others with weak small-world propensity. A network with extremely high small-world features will have a value of SWP approaching unity.

We have found that for low temperatures (T<220), the networks have SWP ≈0.4< SWP${}_{Th}=0.6$, and are in a non-small-world regime. For high temperatures (T>285), the networks have SWP > SWP${}_{Th}=0.6$, and are in a small-world regime. In particular, at room temperature, the networks exhibit a high value of SWP =0.82. In between these two intervals, there is an intermediate regime in which, as temperature rises from 230 to 285 K, the SWP increases, approaching the threshold value SWP${}_{Th}=0.6$ from below. A transition to a small-world regime seems to emerge at 230 K <T< 285 K.

The fact that the small-world property emerges and begins to become dominant as temperature increases, together with the general fact that small-world structure enhances transport in many systems, has inspired us to test whether or not there is any relationship between carrier mobility and the emergent small-world structure. In this respect, using parameters obtained from the network simulations, we have obtained an equation for the hopping carrier mobility μ which, although approximated (because of the finite size of the networks), exhibits nonetheless the well-known dependency $\ln\left(\mu /\mu_{0,LCC}^{*}\right)\propto T^{-2}$. The comparison of this dependence with the one of the SWP metrics on temperature suggests that there is a parallelism between the quick growth of mobility with temperature and the emergence of the small-world property with increasing temperature.

## Data Availability

Not applicable.

## Abbreviations

The following abbreviations have been used in this manuscript:

ARP	Average Return Probability.
CTQW	Continuous-Time Quantum Walks.
DOS	Density of States.
GDM	Gaussian Disorder Model.
MA	Miller–Abrahams.
NS	Network Science.
ODS	Organic Disordered Semiconductor
OFET	Organic Field-Effect Transistors.
OLED	Organic Light-Emitting Diodes.
OS	Organic Semiconductors.
OTFT	Organic Thin-Film Transistors.
QT	Quantum Transport.
RGG	Random Geometric Graph.
RN	Random Network.
RW	Random Walk.
SW	Small-World.
SWP	Small-World Propensity.
SN	Spatial Networks.
TE	Transport Energy.
VRH	Variable-Range Hopping.
**Symbol**	**Definition or Meaning**
A	Adjacency matrix of a graph G.
$a_{ij}$	Element of the adjacency matrix A
D	Node degree matrix: $\mathrm{diag}(k_{1},\cdots ,k_{N})$. It is the diagonal matrix formed from the nodes degrees.
$d_{E}\left(i,j\right)$	Euclidean distance between any pair of nodes *i* and *j* in a network.
$d_{ij}$	Distance between two nodes *i* and *j*. It is the length of the shortest path (geodesic path) between them, that is, the minimum number of links when going from one node to the other.
$E_{F}$	Fermi level.
$\varepsilon_{i}$	Energy of state *i*.
$\varepsilon_{T}$	Transport energy.
$\eta_{HT}$	Hopping transport efficiency.
G	Graph G≡G(N,L,W), where N is the set of nodes (card(N)=N), L is the set of links, and W is weighted adjacency matrix that emerges from our method to link formation.
$\gamma_{0}$	Attempt-to-escape frequency.
$\Gamma_{ij}$	Miller–Abrahams hopping rate between states *i* and *j*.
|i〉	Ket vector in the Hilbert space H. It corresponds to the electron wave function in nanostructure (≡ site ≡ node ≡ ket) *i*.
〈i|	Bra vector in the *dual space* corresponding to the ket |i〉 ∈H
〈k〉	Average node degree.
$k_{B}T$	Thermal energy.
$k_{i}$	Degree of a node *i*. It is the number of links connecting *i* to any other node.
*ℓ*	Average path length of a network. It is the mean value of distances between any pair of nodes in the network.
L	Set of links (edges) of a network (graph).
L	Laplacian matrix of a graph G.
*M*	Size of a graph G. It is the number of links in the set L.
μ	Carrier hopping mobility.
$\mu_{0,LCC}^{*}$	Carrier hopping mobility at low temperature and low carrier concentration.
*n*	Carrier concentration.
*N*	Order of a graph G=(N,L). It is the number of nodes in set N, that is the cardinality of set N: $N=\left|N\right|\equiv \mathrm{card}\left(N\right)$.
N	Set of nodes (or vertices) of a graph.
$N_{S}$	Site concentration.
$p_{j↭k}$	Probability for an electron to evolve between kets |j〉 and |k〉 in the time interval *t*.
$\bar{p}\left(t\right)$	Average return probability
P(k)	Probability density function giving the probability that a randomly selected node has *k* links.
σ	The energy scale of the Gaussian DOS.
W	Weighted adjacency matrix.
$\xi_{\mathrm{loc}}$	Carrier localization length.
